# WeBCMD: A cross-platform interface for the BCMD modelling framework

**DOI:** 10.12688/wellcomeopenres.12201.1

**Published:** 2017-07-26

**Authors:** Joshua Russell-Buckland, Matthew Caldwell, Ilias Tachtsidis

**Affiliations:** 1 Department of Medical Physics and Biomedical Engineering, University College London, London, WC1E 6BT, UK

**Keywords:** mathematical modelling, web technology, cloud platform, NIRS

## Abstract

Multimodal monitoring of the brain generates a great quantity of data, providing the potential for great insight into both healthy and injured cerebral dynamics. In particular, near-infrared spectroscopy can be used to measure various physiological variables of interest, such as haemoglobin oxygenation and the redox state of cytochrome-c-oxidase, alongside systemic signals, such as blood pressure. Interpreting these measurements is a complex endeavour, and much work has been done to develop mathematical models that can help to provide understanding of the underlying processes that contribute to the overall dynamics. BCMD is a software framework that was developed to run such models. However, obtaining, installing and running this software is no simple task. Here we present WeBCMD, an online environment that attempts to make the process simpler and much more accessible. By leveraging modern web technologies, an extensible and cross-platform package has been created that can also be accessed remotely from the cloud. WeBCMD is available as a Docker image and an online service.

## Introduction

Understanding data collected when measuring a biological system can be a complex process. One approach to aid understanding is the use of mathematical models. These can be used alongside the measured data to try and understand the underlying processes that contribute to the observed dynamics. Whilst a lot of time is spent in developing these models, the accessibility and usability of them is often forgotten.

UCL’s Biomedical Optics Research Laboratory has previously developed two software interfaces for defining and solving complex physiological models, BrainCirc ^1, 2^ and BCMD (Brain/Circulation Model Developer) ^3^. While the latter provides simplified modelling and improved stability and portability, it remains challenging to install and use, with some facilities, such as parameter optimisation, only easily available on Linux. In addition, the requirement to create detailed input specification files to define simulation parameters constitutes a significant barrier to use by non-technical specialists.

WeBCMD is an attempt to solve both of these problems by packaging the BCMD framework inside a Docker container and accessing it via a web browser. In taking this approach it has also been possible to develop the interface to access a “cloud service” version of the framework, which is run remotely and managed by the research team. This allows for non-specialists to simply log on to a website and use the software without having to download anything.

### Previous models

It is important to briefly outline the models for which WeBCMD is intended for use. These models are used to help interpret near infrared spectroscopy (NIRS) measurements of the brain.

The BCMD modelling framework was developed to run a number of different mathematical models of brain haemodynamics and metabolism. These models are often extremely complex and do require a reasonable amount of domain knowledge to understand. It is recommended that if you are not familiar with these models that you read their relevant papers in order to better understand them.

**Figure 1.**
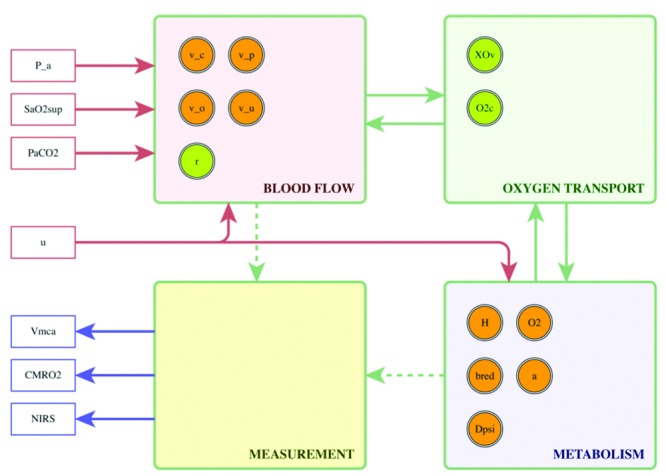
A simplified representation of the BrainSignals structure, as per Caldwell *et al.* ^3^.

**BrainSignals.** The WeBCMD framework will focus on using the BrainSignals model ^4^ and its derivatives. BrainSignals is itself a simplification of the earlier model BrainCirc ^2^, also adding the ability to model metabolism. All of the BrainSignals derived models retain its general structure, with many of the inputs and outputs remaining the same, with small variations to allow for model specific additions, such as scalp blood flow. A simplification of this structure is shown in Figure 1. There are four constituent submodels - blood flow, oxygen transport from blood to tissue, oxidative metabolism within the tissue, and measurement - with a number of state variables passing information between them. For more information on these, please see Banaji *et al.* ^4^.

**Derivatives.** A number of derivative models have been built on top of BrainSignals, such as the simplified BSRV ^3^, which reduced the number of variables and parameters within the model by making various simplifications. This was then developed further to include a scalp submodel and used to investigate the potentially confounding effect of systemic physiological factors on NIRS measurements ^5^.

The model was also developed for use in interpreting data from studies using a piglet model, a common surrogate for the neonatal brain. The models based around this data are often called BrainPiglet models ^6^. Amongst other things, they have been used to investigate the effects of hypoxic-ischemia on the brain ^7^, including functionality to simulate cell death due to oxygen deprivation.

## Methods

### Implementation

WeBCMD was designed to make the BCMD software easily accessible across all operating systems. The software included a number of external dependencies that were either difficult to satisfy or entirely unavailable on Windows and Mac computers. The solution to this was to wrap the entire process inside a Docker container, with all necessary installation steps being handled by the Dockerfile when building the image. This however removed access to the original graphical interface.

Providing native cross-platform GUI support within a Docker application is impractical. Instead, a web-based interface was developed, using a Python web server to mediate the mathematical modelling. As well as supporting cross-platform use locally, this has the additional benefit of allowing the models to be made available in ‘the cloud’.

**Figure 2.**
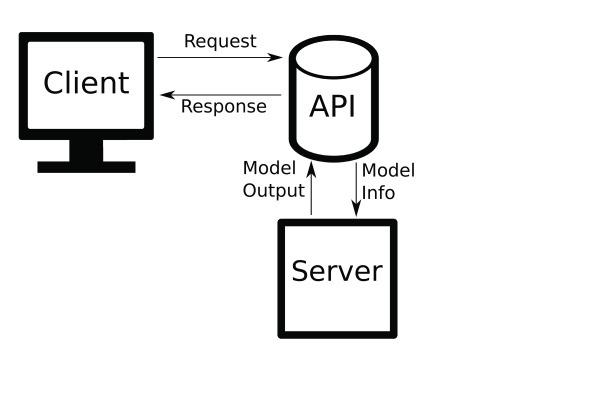
A simplified representation of a web API.

WeBCMD is built primarily around the idea of a "representational state transfer application programming interface" or REST API ^8^. A ‘request’ is sent from the ‘client’ to ‘server’, which handles the request before sending back a ‘response’, as seen in Figure 2.

REST systems of varying complexity are ubiquitous across the internet. The form used in WeBCMD is quite basic, serving simply to separate the interface from the mathematical modelling. Doing so makes the interface easily extensible through the addition of new API routes, while ensuring that the command line interface remains usable.

The client side component of the interface is written entirely in HTML, CSS and javascript and is compatible with any modern web browser. Frameworks, such as Bootstrap and AngularJS, which provide a modern and user friendly design, are easily integrated. The server side component uses the Flask micro webframework, written in Python. It uses API routes to interface with the BCMD modelling software, processing JSON requests sent from the browser, then returning model information and outputs.

Both client-side and server-side components are distributed and installed using Docker, with the files required to build and run the container separate to the WeBCMD code itself. Because the container will run a Linux-based OS, pulling directly to this rather than to the host computer first can avoid problems with different line endings on Windows and Unix-based computers.

To build the Docker container, Docker will need to be installed [ Docker documentation], and if using Linux, Docker Compose will also need installing separately [ Docker Compose documentation] - it is already included in Docker for Windows and Docker for Mac. Once these have been installed, the WeBCMD Docker files can be downloaded in zipped form or cloned from the GitHub repository. The latter allows for updates to be easily pushed and installed using Git. After downloading, navigate to the directory using a terminal emulator such as CMD or Powershell on Windows, Terminal on Mac or xterm on Linux. Build the Docker container using the command:

docker-compose up

which will also load all environment variables.

There are two Docker containers used in the local distribution: the WeBCMD container, which is the same as that used for the online version, and a MongoDB container. The latter is used to store information about models, such as parameter values and default inputs and outputs. It is expected that over time other features will be added that utilise this database, such as detailed descriptions of the physiological significance of each model variable.

***Other uses.*** There are some features that have not yet been fully implemented within the graphical interface, such as sensitivity analysis and model optimisation, and existing users of the software may wish to access these. Whilst these features will not be explored in detail within this paper, this functionality can still be accessed inside the Docker container by using the command line.

To build and launch the container to do this, a slightly different process is required.

1. **Build.** The container needs to be built before running, and the following command needs to be run from inside the directory within the Dockerfile, which ensures that the most recent version of each intermediate container is used:
docker build --no-cache --rm -t webcmd:latest.
2. **Running.** When using the command line interface, if you want to access and store data on the host computer, you will need to use the ‘working’ directory with the following command. Any data you wish to use with the models must be stored in the host_data directory. These can be individual files or child directories.
docker run -it -v /home/user/path/to/host_data:/BCMD/working --entrypoint /bin/bash webcmd

### Operation

The WeBCMD software is available via two main methods: accessing the software via “the cloud", by visiting the WeBCMD website or by downloading the software and running it from inside the Docker container. It is also possible, though not recommended, to run the interface directly using Python. This is most likely to work if the operating system used is a Unix variant, as this is the operating system used within the Docker container.

If accessing the online version of the software, all models and their associated information have already been compiled and stored. The user needs only to follow the on screen steps.

If using the local distribution, the installation steps outlined above will need to be followed. Once this has been done and the containers built and launched, models must be compiled and their information uploaded to the local database. This can be done by accessing the Admin panel in the navigation menu. The local distribution has its own admin user and password that give access to this section. They are

**USER:**    LOCAL_USER

**PASSWORD:**    LOCAL_PASSWORD

Inside the admin section of the interface there are the options to compile a model and to upload model information. Models will need compiling before information can be uploaded, as the model information is generated by running the model itself. Once this has been done, returning to the ‘Home’ tab allows users to review the models that have been compiled and to run them.

## Use cases

There are two distinct use cases for the WeBCMD software at present:

1. Running a model
2. Comparing steady state simulations of autoregulation against a default BrainSignals run

## Running a model

**Figure 3.**
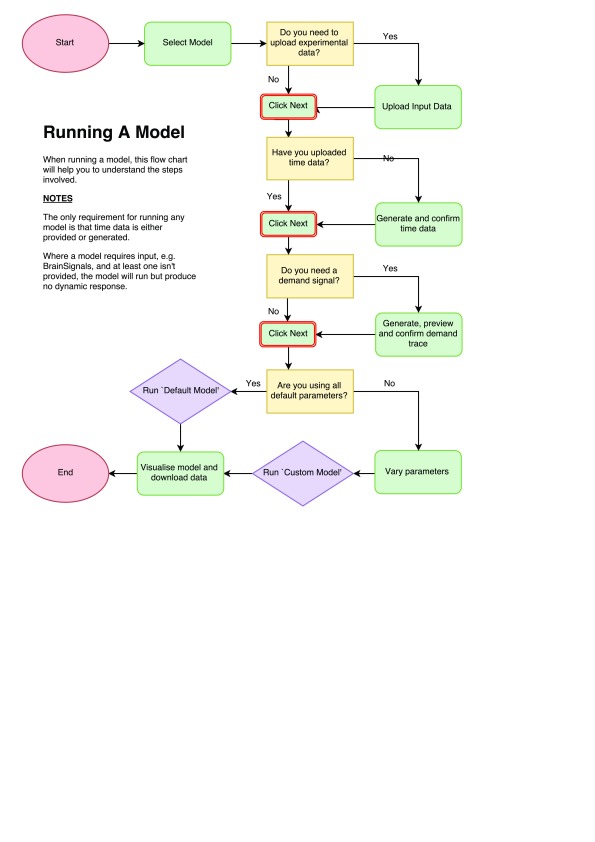
Model run process.

There are 7 main steps to running a model in WeBCMD, once it has been compiled and its data uploaded. Figure 3 shows the general process of running a model in the WeBCMD interface. We will outline this in three specific use cases:

1. A Lotka-Volterra model that requires no input data, run with the default parameters and model inputs and outputs;
2. Driving a BrainSignals model with systemic data, with non-default parameter values;
3. Simulating a number of functional activation tests in BrainSignals, using the demand function generator to create this demand data.

***Lotka-Volterra.*** *The Lotka-Volterra model outlined here is available as part of the WeBCMD distribution. However, solving models such as this is not the intended use of WeBCMD, and there are likely more efficient methods of running and investigating such a model.*

The Lotka-Volterra model is a commonly used model for Predator-Prey dynamics in ecology ^9^. The version shown here consists of two equations

$$\begin{array}{l} \frac{\text{d}x}{\text{d}t}=ax-bxy\\ \frac{\text{d}y}{\text{d}t}=cxy-dy \end{array}$$

where *x* represents the number of prey in the system, *y* represents the number of predators in the system, *t* is time and *a*, *b*, *c*, *d* are positive, real parameters representing the interactions between the two species.

This model will be simulated using a ‘Default Run’, where all parameters are set to their default values and any changes made to them by the user will not be included in the simulation.

The seven main steps to running this model are:

1. Select ‘Lotka-Volterra’ in the model select screen.
2. No data is uploaded, so simply click next.
3. Generate time data here by choosing start and end times, as well as the desired time step, before clicking ‘Generate Time’. Once the time data has been generated ‘t’ will appear in the inputs list at the top of the screen.
4. Demand is BrainSignals specific and so is not required. De-select the checkbox here before clicking next.
5. Because we are using default parameters, simply advance to the next page without altering any values.
6. On the ‘Default’ tab, choose our time point data, which in this case is t. All inputs and outputs are the defaults found in the original model definition. Clicking ‘Run Model’ will send the information to the backend of the interface. When the model output has been returned it will then be possible to click ‘Next’.
7. On this final screen it is possible to visualise and download the returned model data as a comma-separated values (.csv) file. This can the be compared to the model-output-LV.csv file.

***BrainSignals.*** The BrainSignals models are a collection of models that represent the physiological dynamics of the brain. The version referenced here is the original BrainSignals model. For this model, input data is used to drive various inputs, such as blood pressure, partial pressure of CO _2_ and arterial oxygen saturation.

There are two different run types demonstrated here. The first uses systemic data to drive the model, whilst the second uses the built in ‘demand creator’ to simulate a number of demand increases.

**Systemic data.** The seven steps to running this simulation are:

1. Select ‘BrainSignals’ in the model select screen.
2. Click “Browse...” and select the supplied data, which has a default name of synthetic_input_data.csv. Select all of the options as inputs, and confirm. You can leave outputs blank.
3. Click ‘Next’ on the time creation screen as this information is provided in the input file.
4. As we are not simulating functional activation here, we don’t need to create a demand trace so just click ‘Next’ here.
5. Alter parameters to whatever values are desired - in this case the supplied output data was generated by increasing the parameter R_autp to 6.
6. On the ‘Custom’ tab, select the time data to be our t data. Then select the appropriate input data for each input parameter: Pa for ‘P_a’, PaCO2 for ‘Pa_CO2’ and SaO2 for ‘SaO2sup’. Leave all output values as their default settings and then set the ‘Burn in’ time to ‘250’ seconds. Click ‘Run Model’ and when the model has finished running click to visualise on the next screen.
7. On this final screen it is possible to visualise and download the returned model data as a comma-separated values (.csv) file. This can the be compared to the model-output-BS-systemic.csv file.

**Changing demand.** The second run type doesn’t use any input data and instead generates a synthetic input demand. The demand created is not physiologically accurate, but is instead used as a way of showing the possibilities of this functionality. It is expected that more demand input types will be added over time.

**Figure 4.**
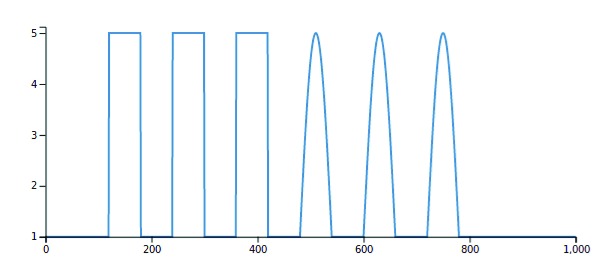
Example demand trace.

1. Select ‘BrainSignals’ in the model select screen.
2. Click ‘Next’ on this screen as no input data is needed.
3. Set the final time to 1000 seconds and click ‘Generate Time’. Once the time data has been created, the inputs section at the top will now contain the letter t. Click ‘Next’.
4. The Demand Creation page will have detected the information from the previous screen and already filled in the Start and End times, as well as the time interval.
  To create a demand peak, click ‘Add Another Peak’. For this example, set the start time for the peak to 120, the end time to 180, the peak height to 5 and select a peak type of ‘Top Hat’. This will create a single square wave with a height of 5 and lasting 60 seconds, starting at 120 seconds. We will repeat this 3 times by clicking repeat peak, typing in 3 for the number of repeats and setting an interval of 60 seconds between each. You can preview this by clicking ‘Generate Demand’.
  We will then add 3 more demand peaks of a different type. Click ‘Add Another Peak’ and enter a start time of 480, end time of 540, peak hight of 5 and select a peak type of ‘Wavelet’. Select ‘Repeat peak’ and repeat it 3 times with an interval of 60 seconds. Click ‘Generate Demand’ and you should get a figure like that in Figure 4. Click ‘Next’.
5. We will leave all parameters at their default values.
6. On the ‘Default’ tab, select the time data, t, to be our ‘t’ data and u to be‘u’. Click ‘Run Model’ and when the model has finished running click to visualise on the next screen.
7. On this final screen it is possible to visualise and download the returned model data as a comma-separated values (.csv) file. This can the be compared to the model-output-BS-demand.csv file.

### Steady state simulations of autoregulation

Steady state simulations of autoregulation are a way of considering the validity of a haemodynamic model like BrainSignals. By altering a single input value, running the model for a period of time and then altering it again, it is possible to compare the autoregulation response of the model to a default data set created using the ‘Default BrainSignals Model’. The input values changed are the arterial blood pressure ( *P _a_*), oxygen saturation ( *Sa*O _2_) and partial pressure of carbon dioxide ( *Pa*CO _2_).

To run a steady state simulation, simply go to the Steady State tab and choose a model. After doing this, you have the option of changing any model parameters, allowing you to visualise their effect on the steady state autoregulation curve. You can then choose whether top run the steady state simulation varying the paramaters in one of three ways:

- **Up**: Vary from min to max
- **Down**: Vary from max to min
- **Both**: Vary from min to max to min, allowing for the detection of hysteresis

Once the model has finished running, going to the next page allows you to visualise the autoregulation curve for this model run alongside the Default BrainSignals curve.

**Figure 5.**
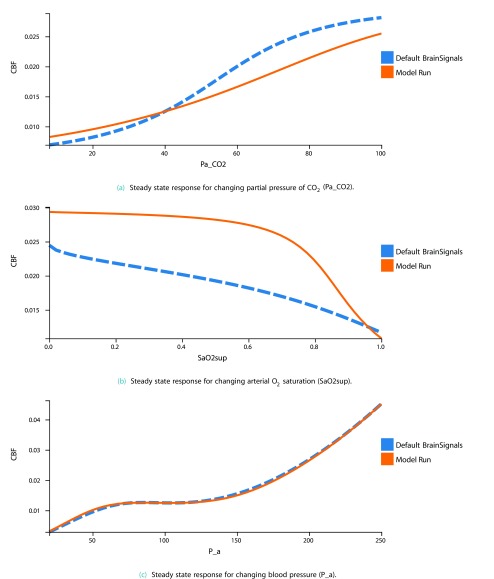
Example steady state output. In blue is the response curve for the default BrainSignals model and in orange is the response curve for this model run’s settings ( R_auto=6).

Figure 5 shows the steady state response curves, when varying from min to max (up), for the BrainSignals model with the R_auto parameter, which is responsible for the autoregulatory reactivity to oxygen, set to 6.

## Summary

When dealing with mathematical models that are themselves complex, it is import to simplify the process of running and analysing these models as much as is possible. If not, there is a risk that the use of the models by non-technical experts will be limited, preventing the potential for insights from the wider scientific community.

WeBCMD has taken the BCMD framework for brain circulation models and made it significantly easier to install and run. The process of installing and managing dependencies can be handled by the framework developers, leaving the process of running and analysing the models to the user. Additionally, the web interface has made input file creation significantly easier by breaking it into easy to follow steps.

The final, and arguably most significant, benefit of the new interface is the cloud based web app. Users who are unable to install the software or simply wish to test the model out before installing are now able to do so by simply accessing a website. The benefits of opening up the modelling software in this manner cannot be overstated. By removing the obvious barrier of installation, the framework and its associated models are now accessible to anyone with access to the internet. This also means that the most recent and stable models will be easily accessible.

## Software and data availability

Web app: http://www.webcmd.org

Source code: https://github.com/buck06191/WeBCMD

Archived source code as at time of publication: WeBCMD is distributed across two repositories. The dockerised distribution can be found at https://doi.org/10.5281/zenodo.832795 ^10^ and is the main source for the software. The dockerised container pulls from a second repository that can be found at https://doi.org/10.5281/zenodo.834661 ^11^. The second repository is not intended for direct use.

License: MIT

All data files are available via Zenodo http://doi.org/10.5281/zenodo.817467 ^12^.
