# Supplementary material for: WeBCMD: A cross-platform interface for the BCMD modelling framework
Source: Wellcome Open Res. 2017 Jul 26;2:56. [Version 1] doi: 10.12688/wellcomeopenres.12201.1 (PMC5571890; doi:10.12688/wellcomeopenres.12201.1)
Supplement: Peer review history

# Peer review: Referee response for version 1

**DOI:** 10.21956/wellcomeopenres.13208.r24522

**Published:** 2017-08-22

**Authors:** Solomon G. Diamond

**Affiliations:** 1 Thayer School of Engineering, Dartmouth College, Hanover, NH, USA

One of the biggest ongoing challenges in neuroimaging research is to accurately interpret measurements in terms of brain physiology and rather than mapping of signal or statistical contrast. Multimodal monitoring of the brain generally and specific tools such as near-infrared spectroscopy help to provide the needed measurements but models of dynamic brain physiology are critically needed for data interpretation. Emerging models of dynamic brain physiology address this need but barriers remain in model implementation for broad dissemination and collaboration in research community. WeBCMD addresses these barriers and has the potential to accelerate scientific advancement in the field.

 WeBCMD in its current form is already a functional and useful research tool. I was particularly pleased to see the steady state simulations of autoregulation since this check of model validity is too often omitted. I agree with the other referee that addition of units for all the parameter values is needed. The variables should also have units and those should be reported in the exported data. Dimensional analysis is one of the key approaches for novices to begin understanding model terms, inputs and outputs and for experts to debug the models.

 My other request is that information be provided about computational load when running online such as memory and computing time. It is quite easy to define models or simulation runs with highly intensive computational demands and so this must always be kept in mind by users and developers alike.

I have read this submission. I believe that I have an appropriate level of expertise to confirm that it is of an acceptable scientific standard.

# Peer review: Referee response for version 1

**DOI:** 10.21956/wellcomeopenres.13208.r24518

**Published:** 2017-08-07

**Authors:** Stephen J. Payne

**Affiliations:** 1 Institute of Biomedical Engineering, Department of Engineering Science, University of Oxford, Oxford, UK

One of the main difficulties in the exploitation of mathematical modelling of physiological systems is the fact that it remains highly challenging to reproduce published results. The problems associated with missing or inconsistent equations and/or parameter values will be well known to every researcher who has attempted to implement a mathematical model from the literature. However, without such models being fully reproducible, it is difficult to use them in any meaningful way. There have been some steps in the direction of model repositories, for example the CellML database (https://www.cellml.org/), which is based on the XML markup language. Despite this, these repositories do require users to download and to learn to use particular software, which can be at different levels of development and require a considerable effort to run. For the dedicated user, such an effort can well be worthwhile; it does, however, make the barrier to entry into the field quite high.

 The approach adopted in this article is thus to try to remove as many of these barriers as possible, which is a thoroughly welcome step. The models that have been implemented have been developed over the last 10-15 years and are now well-established in the field of cerebral blood flow and metabolism. They can be used with confidence, and the equations and parameter values are presented clearly both here and in the literature.

 I used the model directly through the webpage (I did not try the alternative approach of downloading it and running it from inside the Docker container). For a first release it is wisely kept deliberately simple and the model parameters are clearly presented with default values. It was extremely easy to use and the simulations were completed very quickly with the very useful feature of being able to download the results in .csv format. It is helpful to have the Lotka-Volterra example so that users can try a very simple model first if desired.

 This is thus an enormously positive and helpful piece of work, smoothing the path to entry very considerably. Any user can run the model directly from the webpage. The only barrier to entry is now being able to understand the physiological model being implemented. It will be extremely helpful in exploiting these models and it would be hoped that in the future other models will be added to develop a 'one-stop shop' for such models. There are a few minor typos that could usefully be ironed out and I am sure that the authors plan to add a few more features in future (top of my request list would be the addition of units for all the parameter values!); however, I applaud the authors for what they have achieved already and look forward to its future development.

I have read this submission. I believe that I have an appropriate level of expertise to confirm that it is of an acceptable scientific standard.
